# Microbiota Alterations in Lung, Ileum, and Colon of Guinea Pigs with Cough Variant Asthma

**DOI:** 10.3390/ijms25042449

**Published:** 2024-02-19

**Authors:** Chongyang Dou, Lin Hu, Xian Ding, Fangfang Chen, Xi Li, Guihua Wei, Zhiyong Yan

**Affiliations:** School of Life Science and Engineering, Southwest Jiaotong University, Chengdu 610031, China; dou@my.swjtu.edu.cn (C.D.); hulin.swjtu.edu.cn@my.swjtu.edu.cn (L.H.); dingxian@my.swjtu.edu.cn (X.D.); chenfangfang@my.swjtu.edu.cn (F.C.); lixixx@my.swjtu.edu.cn (X.L.)

**Keywords:** cough variant asthma, 16S rDNA, pulmonary microbiota, ileal microbiota, colonic microbiota, interleukin-12, interleukin-13

## Abstract

Alterations in the microbiota composition, or ecological dysbiosis, have been implicated in the development of various diseases, including allergic diseases and asthma. Examining the relationship between microbiota alterations in the host and cough variant asthma (CVA) may facilitate the discovery of novel therapeutic strategies. To elucidate the diversity and difference of microbiota across three ecological niches, we performed 16S rDNA amplicon sequencing on lung, ileum, and colon samples. We assessed the levels of interleukin-12 (IL-12) and interleukin-13 (IL-13) in guinea pig bronchoalveolar lavage fluid using the enzyme-linked immunosorbent assay (ELISA). We applied Spearman’s analytical method to evaluate the correlation between microbiota and cytokines. The results demonstrated that the relative abundance, α-diversity, and β-diversity of the microbial composition of the lung, ileum, and colon varied considerably. The ELISA results indicated a substantial increase in the level of IL-13 and a decreasing trend in the level of IL-12 in the CVA guinea pigs. The Spearman analysis identified a correlation between *Mycoplasma*, *Faecalibaculum*, and *Ruminococcus* and the inflammatory factors in the CVA guinea pigs. Our guinea pig model showed that core microorganisms, such as *Mycoplasma* in the lung, *Faecalibaculum* in the ileum, and *Ruminococcus* in the colon, may play a crucial role in the pathogenesis of CVA. The most conspicuous changes in the ecological niche were observed in the guinea pig ileum, followed by the lung, while relatively minor changes were observed in the colon. Notably, the microbial structure of the ileum niche approximated that of the colon niche. Therefore, the results of this study suggest that CVA development is closely related to the dysregulation of ileal, lung, and colon microbiota and the ensuing inflammatory changes in the lung.

## 1. Introduction

Cough variant asthma (CVA) is considered an asthma subset in which chronic cough is the only symptom [1]. However, it is accompanied by mild bronchial dilation, airway remodeling, and increased bronchial reactivity [2,3,4,5]. CVA is influenced by both genetic and environmental factors, and its etiology is complex and differs from typical allergen-induced asthma [6,7]. Although the exact pathophysiological mechanisms of CVA are not clear, recent studies indicate that the microbiota in the lung and gut may be potential factors in the development and worsening of asthma symptoms [8].

The gut and respiratory tract of the organism are home to trillions of microorganisms collectively known as the microbiota. These microorganisms play a crucial role in maintaining host health and immunity [9,10]. The microbiota of the gut and respiratory tract is widely recognized as complex and dynamic communities that interact with the host’s immune system and other host factors [11,12]. Studies have shown that changes in microbial composition, or so-called dysbiosis, can lead to various diseases, including asthma [13,14]. To date, research on the gut microbiota has been more extensive. Non-breastfed newborns and infants born via cesarean section are at risk of dysbiosis in the gut microbiota, which can lead to allergic diseases [15]. Studies have also found that there is a critical window of time for gut colonization in germ-free mice to limit TH2-mediated allergic reactions [16]. In addition, the lungs, with an independent microbial ecosystem, have their unique microbiota. The respiratory microbiota enters the lungs through micro-aspiration of respiratory and pharyngeal secretions and is the primary source of the healthy human pulmonary microbiome [17]. Dysregulation of the lung microbiota affects the local accumulation of Treg cells, leading to susceptibility to allergic airway inflammation [18].

Although much of asthma research has focused on the role of gut microbiota, evidence suggests that respiratory microbiota may also play an important role in the development of allergic asthma [19,20]. Dysbiosis in the ecosystems of the respiratory tract and gut may lead to chronic inflammation and may be associated with CVA development. Currently, guinea pigs are commonly used to study the potential mechanisms of asthma development [21]. In this study, we aim to investigate the microbial composition of the lung, ileum, and colon in CVA guinea pigs and compare it to normal guinea pigs. These findings may help develop new strategies for the prevention and treatment of CVA.

## 2. Results

### 2.1. The Changes in Inflammatory Factors of Guinea Pigs

**Figure 1:**
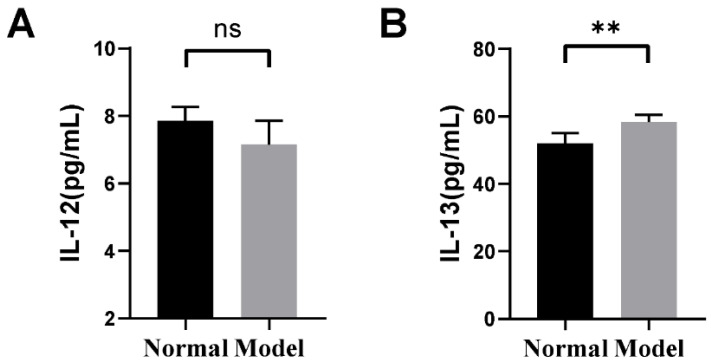
Changes in BALF inflammatory factors in guinea pigs. (**A**) Changes in interleukin 12 in BALF. (**B**) Changes in interleukin 13 in BALF. ** *p* < 0.01, compared to the normal group. The ns indicates that no statistical difference was observed between the two groups. Data are expressed as the mean ± SD (*n* = 6/group). The black column represents the normal group. The grey column represents the model group.

Based on the respective ELISA assay kits, the levels of IL-12 and IL-13 in the BALF of each guinea pig group were assessed. The results indicated that the level of IL-12 in the model group exhibited a decreasing trend compared to the normal group (Figure 1A). Furthermore, the level of IL-13 was significantly elevated in the model group compared to the normal group (Figure 1B).

### 2.2. Metadata and Sequencing of Microbiota

**Figure 2:**
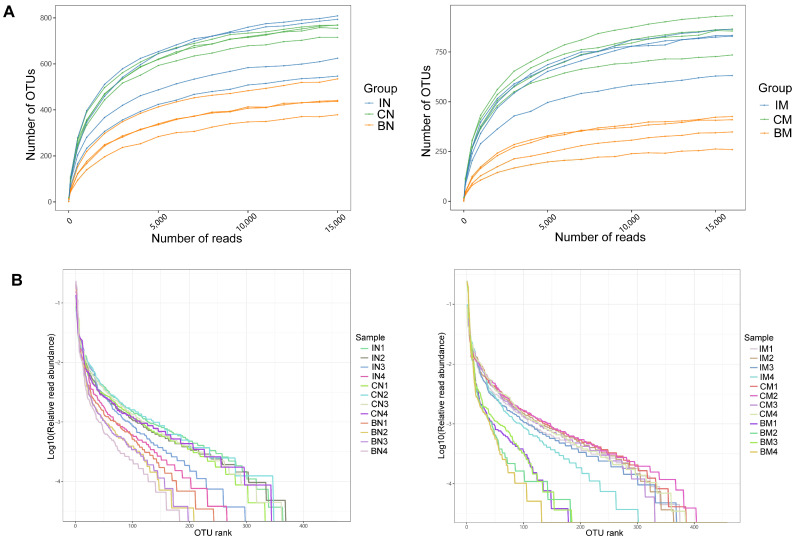
Rarefaction Curve and Rank−Abundance curve of all samples. (**A**) The Rarefaction Curve shows the trend of changes in species richness of each sample with sequencing depth. (**B**) The Rank−Abundance curve displays the relationship between the abundance of individual species and the number of individual species in each sample. IN (the microbiota in the ileum of the normal group), CN (the microbiota in the colon of the normal group), BN (the microbiota in the BALF of the normal group), IM (the microbiota in the ileum of the CVA model group), CM (the microbiota in the colon of the CVA model group), BM (the microbiota in the BALF of the CVA model group). Guinea pigs were randomly divided into a normal group and a model group, with six guinea pigs in each group. Sequencing analysis was conducted on four samples from each group.

We filtered out low-quality and chimeric sequences from 24 samples and obtained a total of 573,815 clean tags. The effective sequences were clustered into operational taxonomic units (OTUs) at a 97% similarity level. In the normal group, the lung contained 583 OTUs, the ileum contained 664 OTUs, and the colon contained 905 OTUs. In the model group, the lung had 567 OTUs, the ileum had 856 OTUs, and the colon had 965 OTUs. By plotting the Rarefaction Curve (Figure 2A) and Rank-Abundance curve (Figure 2B), the richness and evenness of species in the samples can be indirectly reflected. The gradual flattening of the curves indicates that the sequencing depth has basically covered all the species in each sample, and the distribution of species in the samples is relatively even.

### 2.3. Evaluation of Microbial Composition in Three Different Ecological Niches of Normal Guinea Pigs

**Figure 3:**
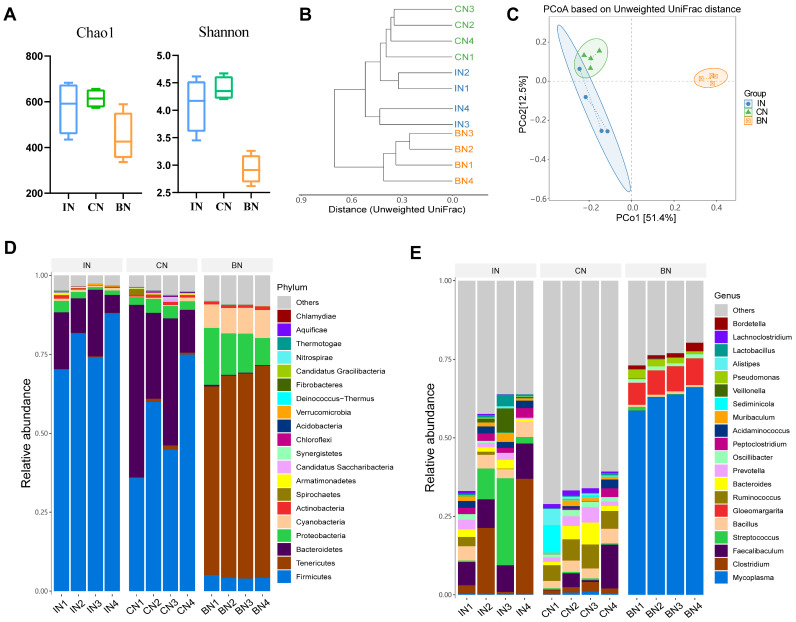
Comparison of microorganisms in three different niches in normal guinea pigs. (**A**) Alpha diversity. (**B**) Clustering tree based on unweighted UniFrac distances. (**C**) PCoA based on unweighted UniFrac. The horizontal axis denotes the first principal coordinate, with percentages indicating the contribution of the first axis to the differences among samples. The vertical axis represents the second principal coordinate, with percentages indicating the contribution of the second axis to the differences among samples. Each point on the graph represents a sample, with samples from the same group represented by the same color. Confidence ellipses indicate the 95% confidence interval for each group. (**D**) Distribution of high-abundance microbial groups at the phylum level, with sample names on the horizontal axis and relative abundance on the vertical axis, and different colors representing different taxa. (**E**) Distribution of high-abundance microbial groups at the genus level, with sample names on the horizontal axis and relative abundance on the vertical axis, and different colors representing different taxa.

To analyze the characteristics of microbiota in three distinct ecological niches, we conducted research on the microbiota in the lungs, ileum, and colon of the normal group. The alpha diversity index is a crucial indicator of the complexity of microbiota in a given sample. It encompasses various aspects of the community, such as richness, evenness, and diversity. Richness can be quantified using the Chao1 index, which reflects the total number of species in the community. On the other hand, diversity can be measured using the Shannon index, which indicates the extent of diversity within the community. As the value of the Shannon index increases, so does the diversity of the community. In the normal group, the Chao1 index of colon samples is the highest and the Shannon index is the largest, indicating that the richness and diversity of the colon microbiota are higher than those of the lung and ileum. Additionally, the richness and diversity of the lung ecological niche are much lower than those of the ileum and colon ecological niches (Figure 3A).

By conducting a clustering analysis on the samples, we can explore the similarity of community composition among multiple different samples. As shown in Figure 3B, samples from the same ecological niche were clustered together, indicating that the community structure of parallel samples within groups was similar. The results showed samples from the ileum and colon were closer and had shorter branches, indicating that the community structure of ecological niches between them was more similar. Subsequently, to characterize the variation between samples, beta diversity analysis was adopted. Firstly, the sample distance was calculated based on the OUT table, and then Principal Coordinates Analysis (PCoA) was used for analysis. PCoA is based on the calculated unweighted UniFrac distances for subsequent analysis, which is more convenient for observing the degree of difference and the changing pattern between samples. Different points in the graph represent different samples, and the greater the distance between points, the greater the difference in community structure. Conversely, the smaller the difference in community structure, the closer the points are. As shown in Figure 3C, the distance between samples within each lung, ileum, and colon group was close, while the relative distance between groups was far, indicating that the community structure of normal guinea pigs’ lungs, ileum, and colon differs greatly. 

Based on the results of the taxonomic analysis, we compared the taxonomic composition of normal guinea pig lung, ileum, and colon microbiota at various classification levels. At the phylum level (Figure 3D), the dominant phyla in the ileum microbiota were *Firmicutes*, *Bacteroidetes*, *Proteobacteria*, *Cyanobacteria*, and *Actinobacteria*, with relative abundances of 78.45%, 13.89%, 1.95%, 0.70%, and 0.51%, respectively. The dominant phyla in the colon microbiota were *Firmicutes*, *Bacteroidetes*, *Proteobacteria*, *Tenericutes*, and *Actinobacteria*, with relative abundances of 53.88%, 33.87%, 3.39%, 0.49%, 0.79%, and 0.78%, respectively. The dominant phyla in the lung microbiota were *Tenericutes*, *Proteobacteria*, *Cyanobacteria*, *Firmicutes*, and *Actinobacteria*, with proportions of 64.00%, 12.95%, 8.13%, 4.4%, and 0.98%, respectively. At the genus level (Figure 3E), the top five abundant genera in the ileum microbiota were *Clostridium* (15.36%), *Streptococcus* (10.02%), *Faecalibaculum* (9.06%), *Bacillus* (4.19%), and *Veillonella* (2.35%). The top five genera in the colon microbiota were *Ruminococcus* (6.26%), *Faecalibaculum* (4.79%), *Bacteroides* (3.49%), *Bacillus* (3.45%), and *Prevotella* (2.69%). The important genera in the lung microbiota were *Mycoplasma* (62.84%), *Gloeomargarita* (7.80%), *Pseudomonas* (1.90%), *Bordetella* (1.54%), and *Rhodopseudomonas* (1.24%).

### 2.4. Microbial Composition of the Ileum of Guinea Pigs Varied Maximally in the Three Different Ecological Niches

**Figure 4:**
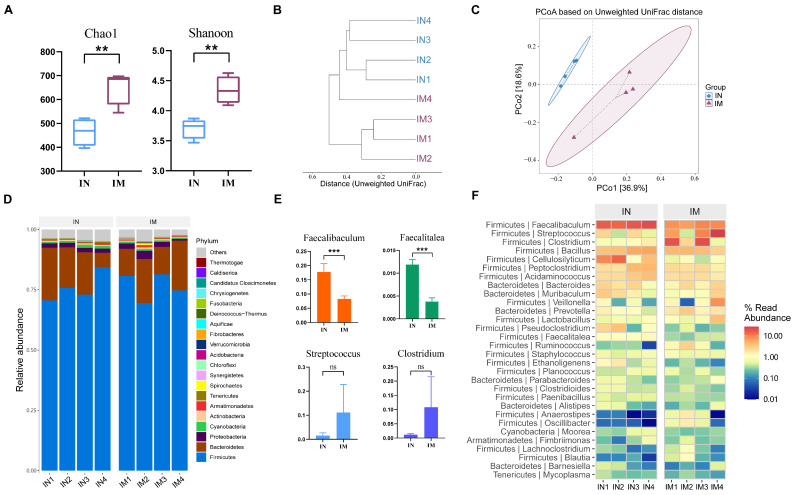
Comparison of ileal microbiota between normal and CVA model groups. (**A**) Alpha diversity. ** *p* < 0.01. (**B**) Clustering tree based on unweighted UniFrac distances. (**C**) PCoA based on unweighted UniFrac. (**D**) Distribution of high-abundance microbial groups at the phylum level, with sample names on the horizontal axis and relative abundance on the vertical axis, and different colors representing different taxa. (**E**) Changes in dominant microbiota at the genus level. *** *p* < 0.001. The ns indicates that no statistical difference was observed between the two groups. (**F**) Heatmap of the top 30 abundant genera selected based on abundance ranking, with sample names sorted on the horizontal axis and the vertical axis arranged according to the total average abundance from top to bottom, making it easier to observe which genera have higher average abundance and how they vary between samples.

We analyzed the changes in the microbiota of the ileum between the normal group (IN) and the model group (IM). Calculation of the alpha diversity index showed a significant increase in Chao1 in the IM group compared to the IN group (*p* < 0.01). The Shannon index of the IM group was significantly higher than that of the IN group (*p* < 0.01, Figure 4A).

To visually compare the similarity between different samples, we utilized clustering analysis for calculation and visualization. The results showed that samples from the same group were closer together and had shorter branch lengths, indicating similar community structures among parallel samples within the same group (Figure 4B). We then investigated the changes in microbial composition and the similarity between samples by calculating the beta diversity of each group (Figure 4C). The distance between samples from the IN and IM groups was far apart, and the composition of their microbiota differed significantly, indicating a significant change in the microbial composition of the ileum in the CVA model guinea pigs compared to the normal group.

To further visualize the species data in the ileum samples, we conducted analysis at the phylum and genus levels (Figure 4D,F). The top five phyla represented in both the IN and IM groups were *Firmicutes*, *Bacteroidetes*, *Proteobacteria*, *Cyanobacteria*, and *Actinobacteria*, accounting for 94.71% and 94.23% of the total, respectively. Compared to the IN group, the relative abundance of *Bacteroidetes* and *Cyanobacteria* decreased, while that of *Firmicutes*, *Actinobacteria*, and *Proteobacteria* increased in the IM group, but none of these changes were significant. At the genus level, *Faecalibaculum*, *Streptococcus*, *Clostridium*, *Bacillus*, and *Cellulosilyticum* were dominant in both the normal and model groups. The abundance of *Faecalibaculum* and *Faecalitalea* was significantly lower in the IM group compared to the IN group (*p* < 0.001, Figure 4E), while the abundance of *Streptococcus* and *Clostridium* was higher in the IM group, but the differences were not significant (*p* > 0.04).

**Figure 5:**
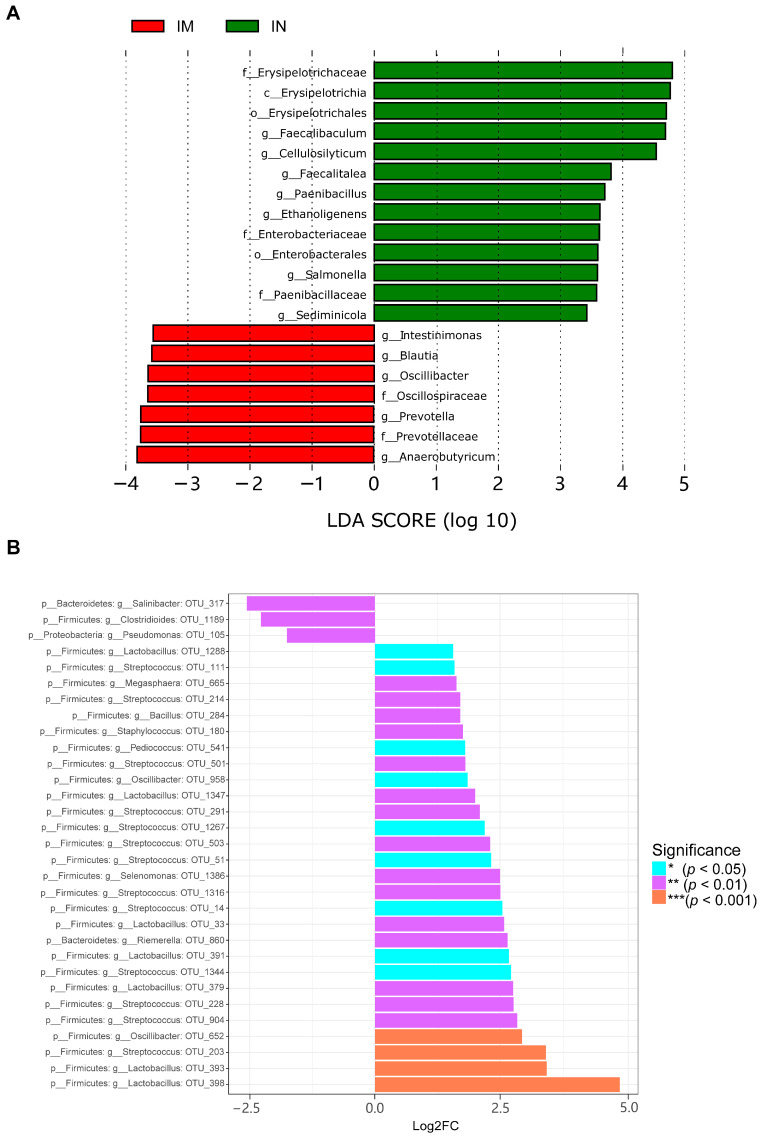
Differential species analysis of the gut microbiota in the ileum. (**A**) The horizontal axis represents the magnitude of the LDA score, where the length of the bar indicates the importance of the corresponding group. The color represents different groups, indicating that these groups have significantly enriched features, with the highest average abundance and significant differences between groups. (**B**) The positive values of the horizontal axis Log2FC represent up-regulated species, while negative values represent down-regulated species, and the vertical axis represents the taxonomic classification of the species, with three columns indicating phylum, genus, and OTU. The color represents the degree of significance.

The analysis of linear discriminant effect size (LEfSe) is utilized to identify significant differences in communities or species between groups. Species with LDA values exceeding the set threshold of >3 are considered biomarkers with statistically significant differences between groups. The results showed that the dominant species with significant differences in the IN group included *Faecalibaculum* and *Faecalitalea*, while the IM group was significantly enriched with *Intestinimonas*, *Blautia*, *Oscillibacter*, and *Anaerobutyricum* (Figure 5A). Additionally, we used the metagenomeSeq method to search for species with statistically significant differences in the gut microbiome associated with CVA (Figure 5B). The results demonstrated that 31 different species of gut microbiota collectively constituted 12 genera. As the exact species of gut microbiota cannot be determined in this study, the OUT value represents the species within the corresponding genus of gut microbiota. Compared to the normal group of guinea pigs, the relative abundance of *Clostridioides* OTU_1189 and *Pseudomonas* OTU_105 decreased in the model group. Conversely, the species exhibiting an increased relative abundance include *Lactobacillus* OTU_398, *Streptococcus* OTU_203, as well as *Bacillus* OTU_284.

### 2.5. Microbial Composition of the Lung of Guinea Pigs Varied Secondarily in the Three Different Ecological Niches

**Figure 6:**
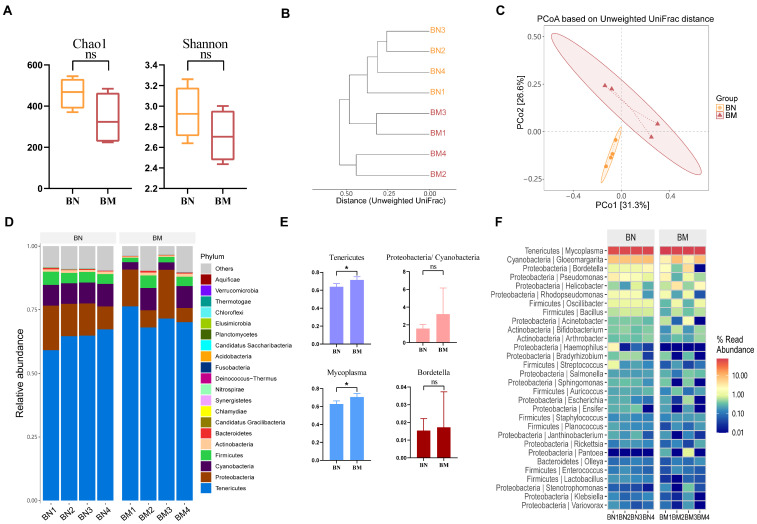
Comparison of pulmonary microbiota between normal and CVA model groups. (**A**) Alpha diversity. (**B**) Clustering tree based on unweighted UniFrac distances. (**C**) PCoA based on unweighted UniFrac. (**D**) Distribution of high-abundance microbial groups at the phylum level. (**E**) Changes in dominant microbiota at the genus level. * *p* < 0.05. The ns indicates that no statistical difference was observed between the two groups. (**F**) Heatmap of the top 30 abundant genera selected based on abundance ranking.

We then analyzed the changes in the microbiota in the BN and BM groups. Although the Chao1 and Shannon indices of the lung microbiota in the model group guinea pigs were decreased compared to the normal group, the differences were not statistically significant (*p* > 0.05, Figure 6A). PCoA showed a significant difference between the lung microbiota of the normal and model group guinea pigs (Figure 6C).

The data on species in the lungs were analyzed to further understand the distribution and composition of microbiota at the phylum and genus levels. At the phylum level, the two groups were mainly composed of *Tenericutes*, *Proteobacteria*, *Cyanobacteria*, *Firmicutes*, and *Actinobacteria*. Compared to the BN group, the BM group had a significantly higher abundance of *Tenericutes*, while other phyla did not show significant changes. It is worth noting that the proportion of *Proteobacteria* and *Cyanobacteria* increased in the BM group compared to the BN group (Figure 6E). At the genus level, the two groups were mainly composed of *Mycoplasma*, *Gloeomargarita*, *Bordetella*, *Pseudomonas*, and *Helicobacter* (Figure 6F). Compared to the BN group, the BM group had a significantly higher abundance of *Mycoplasma* (Figure 6E), but the increase in *Bordetella* was not significant.

**Figure 7:**
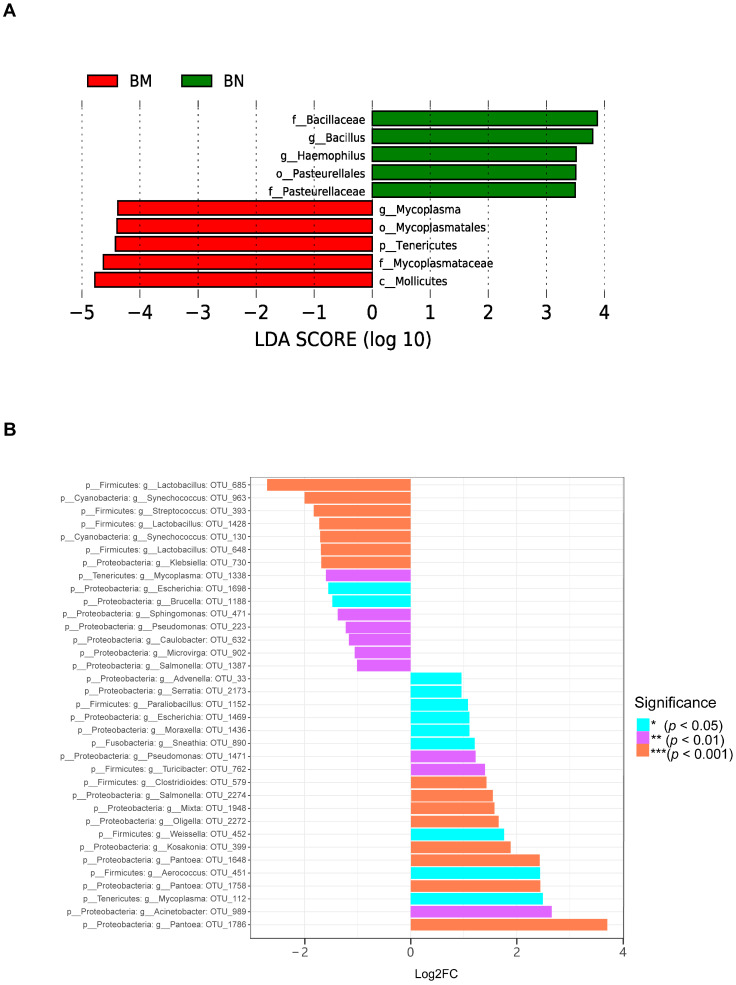
Differential species analysis of the lung microbiota. (**A**) The horizontal axis represents the size of the LDA score, with longer bars indicating more important taxa. The colors represent different groups, indicating that these taxa are significantly enriched in this group, with the highest average abundance and significant inter-group differences. (**B**) The horizontal axis represents the Log2FC value, with positive values indicating up-regulated species and negative values indicating down-regulated species. The vertical axis represents the taxonomic attributes of species, with three columns representing phylum, genus, and OTU. The colors represent the level of significance.

We also used LEfSe analysis to find biomarkers that are important and significantly different between the BN and BM groups. The results showed that *Mycoplasma*, *Mycoplasmatales*, *Tenericutes*, and *Mycoplasmataceae* were significantly different in BM (Figure 7A). Using the metagenomeSeq method, we identified 35 different microbial species that constituted 26 bacterial genera (Figure 7B). Among them, down-regulated species included *Lactobacillus* OTU_685, *Streptococcus* OTU_393, and *Mycoplasma* OTU_1338, while up-regulated species included *Pantoea* OTU_1786, *Acinetobacter* OTU_989, and *Mycoplasma* OTU_112.

### 2.6. Microbial Composition of the Colon of Guinea Pigs Varied Minimally in the Three Different Ecological Niches

**Figure 8:**
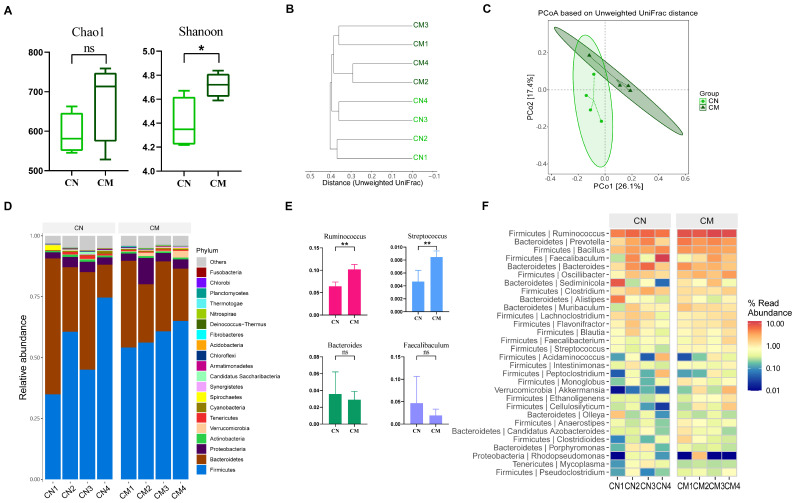
Comparison of colonic microbiota between normal and CVA model groups. (**A**) Alpha diversity. * *p* < 0.05. (**B**) Clustering tree based on unweighted UniFrac distances. (**C**) PCoA based on unweighted UniFrac. (**D**) Distribution of high-abundance microbial groups at the phylum level. (**E**) Changes in dominant microbiota at the genus level. ** *p* < 0.01. The ns indicates that no statistical difference was observed between the two groups. (**F**) Heatmap of the top 30 abundant genera selected based on abundance ranking.

To assess the impact of CVA on the systematic evolutionary composition of colon microbiota, we compared the microbiota of CN and CM groups. The results of alpha diversity showed that the Chao1 index of the colon in the model group increased, with no significant difference in variation (*p* > 0.05), but the Shannon index significantly increased (*p* < 0.05, Figure 8A). The PCoA based on unweighted UniFrac distances showed that there was partial overlap in the confidence ellipses between the colon normal group and the model group (Figure 8C). Therefore, we further validated whether there were significant differences in sample distance between the CN and CM groups using PerMANOVA. The results showed that there was no significant difference between the CN and CM groups (R2 = 0.20955, *p* = 0.085 > 0.05).

To illustrate the trend of changes in high-abundance species at the phylum level, a Sankey diagram was used for community composition analysis. The most common phyla in CN and CM groups were *Firmicutes*, *Bacteroidetes*, *Proteobacteria*, *Actinobacteria*, and *Verrucomicrobia* (Figure 8D). Compared to the normal group, the model group showed an increase in *Firmicutes* and *Proteobacteria* and a decrease in *Bacteroidetes* in colon samples, leading to a decrease in the ratio of *Bacteroidetes*/*Firmicutes*. At the genus level, *Ruminococcus*, *Prevotella*, *Bacillus*, *Faecalibaculum*, and *Bacteroides* were dominant genera in both CN and CM groups (Figure 8F). The abundance of *Ruminococcus* in the CM group was significantly higher than that in the CN group. In addition, compared to the normal group, the relative abundance of *Faecalibaculum* and *Bacteroides* decreased in the model group. We also found that the relative abundance of *Streptococcus* significantly increased in colon samples of the model group compared to the normal group (Figure 8E).

**Figure 9:**
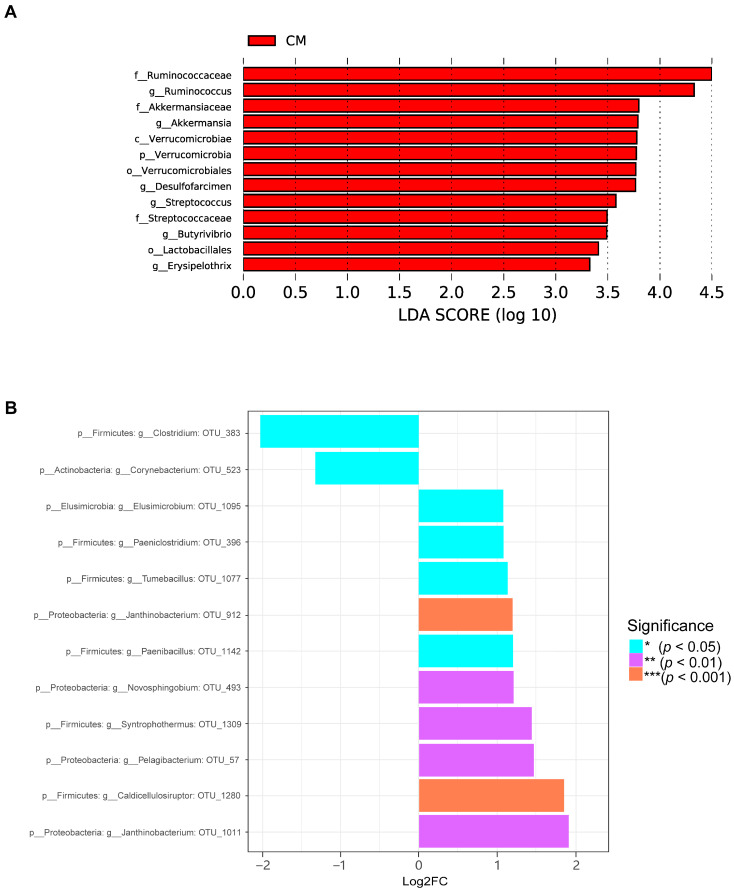
Differential species analysis of the gut microbiota in the colon. (**A**) The horizontal axis represents the size of the LDA score, with longer bars indicating more important taxa. The colors represent different groups, indicating that these taxa are significantly enriched in this group, with the highest average abundance and significant inter-group differences. (**B**) The horizontal axis represents the Log2FC value, with positive values indicating up-regulated species and negative values indicating down-regulated species. The vertical axis represents the taxonomic attributes of species, with three columns representing phylum, genus, and OTU. The colors represent the level of significance.

Subsequently, LEfSe analysis revealed the differentially enriched microbial genera in the CM group, including *Ruminococcaceae*, *Ruminococcus*, *Akkermansia*, and *Streptococcus* (Figure 9A). Using the metagenomeSeq method, we identified 12 different microbial species that constituted 11 microbial genera (Figure 9B). Among them, down-regulated species included *Clostridium* OTU_383 and *Corynebacterium* OTU_523, while up-regulated species included *Janthinobacterium* OTU_1011, *Caldicellulosiruptor* OTU_1280, and *Pelagibacterium* OTU_57.

### 2.7. Evaluation of Microbial Composition in Three Different Ecological Niches of CVA Guinea Pigs

**Figure 10:**
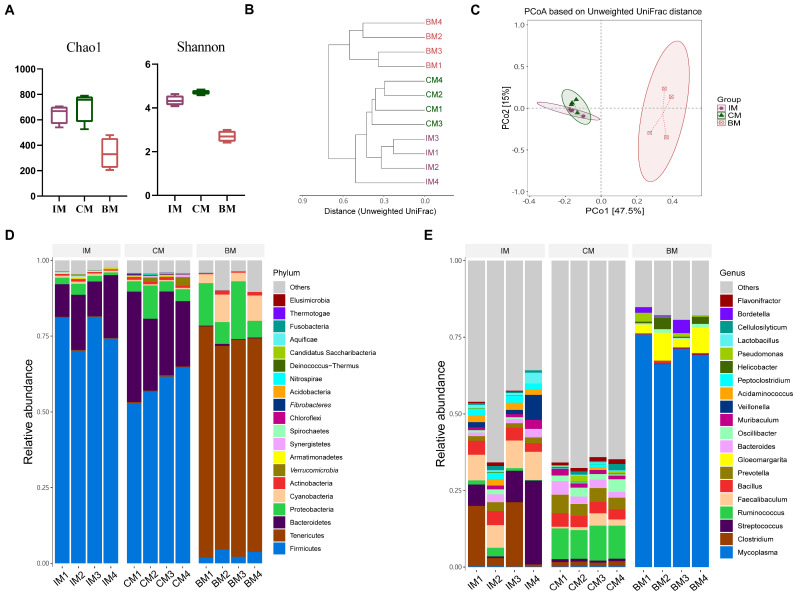
Comparison of microorganisms in three different niches in CVA model guinea pigs. (**A**) Alpha diversity. (**B**) Clustering tree based on unweighted UniFrac distances. (**C**) PCoA based on unweighted UniFrac. (**D**) Distribution of high-abundance microbial groups at the phylum level. (**E**) Distribution of high-abundance microbial groups at the genus level.

We further investigated the characteristics of the microbiota in three different ecological niches in the CVA model group to explore the relevance between them. In the CVA model, although both the Chao1 and Shannon indices in the ileum significantly increased, the colon remained with the highest alpha diversity index among the three ecological niches (Figure 10A). Cluster analysis based on the unweighted UniFrac distances matrix revealed that the structure of the ileal microbiota in the CVA model group was closer to that of the colonic microbiota than that of the normal group (Figure 10B). The beta diversity results showed that the microbial composition in the lung samples was still significantly different from that in the ileum and colon. However, it is worth noting that the microbial structure of the ileum and colon was similar (Figure 10C). This suggests that the differences between the microbiota in the ileum and colon were relatively small in the CVA guinea pigs.

We analyzed the relative abundance of microbiota in the phylum and genus to explore the microbial composition of the three ecological niches in CVA guinea pigs. At the phylum level (Figure 10D), *Firmicutes*, *Bacteroidetes*, *Proteobacteria*, *Cyanobacteria*, and *Actinobacteria* accounted for a total of 95.38% of the ileal microbiota; the colonic microbiota, *Firmicutes*, *Bacteroidetes*, *Proteobacteria*, *Verrucomicrobia*, and *Actinobacteria* accounted for 93.67% of the total; and among the lung microbiota, *Tenericutes*, *Proteobacteria*, *Cyanobacteria*, *Firmicutes*, and *Actinobacteria* accounted for a total of 92.58%. At the genus level (Figure 10E), the dominant genera in the ileum included *Streptococcus*, *Clostridium*, *Faecalibaculum*, *Bacillus*, and *Veillonella*; the dominant genera in the colon included *Ruminococcus*, *Prevotella*, *Bacillus*, and *Bacteroides*. The dominant genera in the lung included *Mycoplasma*, *Gloeomargarita*, *Helicobacter*, *Bordetella*, and *Pseudomonas*.

### 2.8. Correlation Analysis of Microbiota and Inflammatory Factors

**Figure 11:**
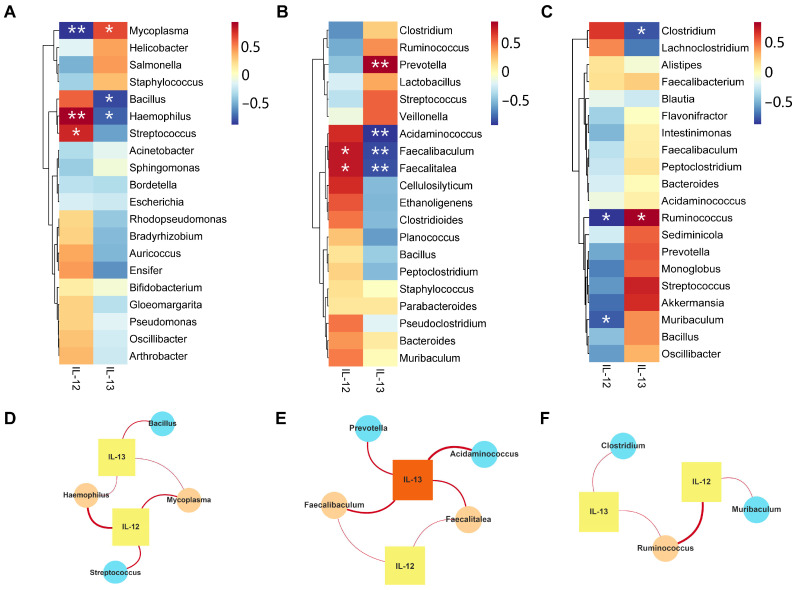
The correlation between microbiota and inflammatory factors. (**A**) Heat map showing the correlation between the top 20 abundant genera of pulmonary microbial communities and lung inflammatory factors. The redder the color, the stronger the positive correlation, while the bluer the color, the stronger the negative correlation. * *p* < 0.05, ** *p* < 0.01. (**B**) Heat map showing the correlation between the top 20 abundant genera of ileal microbial communities and lung inflammatory factors. (**C**) Heat map showing the correlation between the top 20 abundant genera of colonic microbial communities and lung inflammatory factors. (**D**) Network diagram depicting the correlation between the top 20 abundant genera of pulmonary microbial communities and lung inflammatory factors. Circular nodes represent microbial communities at the genus level, while square nodes represent inflammatory factors. The degree indicates the number of edges connected to the node, and a larger degree indicates that the species is more central in the network. The species point size is correlated with degree, the color of the smallest species point value is light blue−green, and the color of the largest selected species point value is light orange. The color with the smallest inflammatory factor score is pale yellow and the color with the largest inflammatory factor point value is orange. (**E**) Network diagram depicting the correlation between the top 20 abundant genera of ileal microbial communities and inflammatory factors. (**F**) Network diagram depicting the correlation between the top 20 abundant genera of colonic microbial communities and inflammatory factors.

We employed the Spearman correlation analysis to delve deeper into the correlation between various microbiota and inflammatory factors in distinct ecological niches. Initially, we explored the link between lung microbiota and BALF inflammatory factors, where we identified certain microbial genera that correlated with BALF inflammation. At the genus level, IL-12 negatively correlated with *Mycoplasma* but positively correlated with *Hemophilus* and *Streptococcus*. IL-13 positively correlated with *Mycoplasma* but negatively correlated with *Hemophilus* (Figure 11A). To expedite the identification of core species closely associated with physical and chemical factors, we constructed a network of points and lines to depict the “social structure” between microbiota and inflammatory factors. Our results revealed that *Mycoplasma* and *Hemophilus* were the core microorganisms closely linked to pulmonary inflammatory factors (Figure 11D). Subsequently, we investigated the relationship between the ileal and colonic microbiota and pulmonary inflammatory factors to further explore the association between specific genera in the gut and inflammatory levels. *Faecalibaculum* and *Faecatalea* in the ileum exhibited a negative correlation with IL-13, whereas they displayed a positive correlation with IL-12 (Figure 11B). It is worth noting that *Faecalibaculum* and *Faecalitalea* were regarded as core microorganisms closely associated with inflammatory factors (Figure 11E). In the colonic microbiota, *Ruminococcus* was positively correlated with IL-13. *Ruminococcus* and *Muribaculum* were negatively correlated with IL-12 (Figure 11C). *Ruminococcus* was a core microorganism at the genus level that was closely associated with inflammatory factors (Figure 11F).

## 3. Discussion

In this study, as the number of OVA aerosol stimulations increased, the model group of guinea pigs exhibited poor mental states and a slow weight gain, and gradually developed symptoms such as coughing, raised hair, an increased secretion of oral and nasal cavities, a difficulty in breathing, and abdominal twitching. These observations are consistent with the findings of Jiao et al. and, in conjunction with the previous studies of our group, indicate that the CVA guinea pig model was successfully constructed in this study [22,23]. Increasing evidence suggests that the imbalance in gut and lung microbiota affects the occurrence and progression of respiratory system diseases [24,25]. The dynamic microbial balance formed between the host and the microbiota usually constitutes an ecological system of interconnection and interaction. The disruption of this balance may lead to the development of various diseases in the host organism [26]. Therefore, in this article, we systematically studied the microbial composition of the lung, ileum, and colon of normal and CVA guinea pigs, and emphasized the unique changes in the microbiota of different parts of guinea pigs, in order to better understand the potential correlation between CVA and microbiota.

By analyzing the alpha diversity of the microbiota in the lung, ileum, and colon of normal guinea pigs, we discovered that the colon had the highest number and diversity of species, while the lungs had the lowest. Similarly, using beta diversity to display differences, there was a significant difference in the microbial structure of the lung, ileum, and colon in the normal group. A large number of microorganisms exist on the various mucosal surfaces of the body, with the majority being present on the surface of gut, followed by other mucosal surfaces, including the lungs [27]. A healthy lung was long believed to be sterile, but with the advent of 16S rDNA sequencing technology, research on lung microbiota has progressed [28]. The flow of microorganisms in the lungs can move in two directions with the airflow, but directional movement and sphincter muscles ensure the unidirectional flow of gut microbiota. The respiratory tract has a limited nutritional supply, while the gut microbiota is rich in nutrients. These characteristics result in a high turnover rate but a low quantity of microbial populations in the lungs, with 103–106 bacteria per gram of tissue compared to 10^11^–10^12^ per gram of tissue in the gut [29]. The results of this study also reflect the phenomenon that the number of microorganisms in the lungs is lower than in the gut. Furthermore, the microbiota present in the healthy human gut exhibits high inter-individual variation, with different communities observable along the length of the gut, indicating significant spatial heterogeneity in the ileum and colon [30]. Oxygen levels, antimicrobial peptides (including bile acids), and pH gradients limit bacterial density in the ileum community [31,32]. Meanwhile, the ileum has a faster peristalsis rate and shorter retention time of intestinal contents, while the colon is the opposite, resulting in a higher number of colon microbiota. The microbiota has been shown to play a role in human health and disease [33]. Therefore, we conducted a systematic evaluation of the respiratory and gut microbiota in health and disease by focusing on the lung, ileum, and colon ecological niches.

Firstly, we analyzed the microbiota of the ileal ecological niche. The species richness, diversity, and evenness of the IM group were significantly different from those of the IN group, and their community structure was also greatly different. This indicates that the ileal microbiota has become dysbiotic. The imbalance in the gut microbiota first damages the intestinal mucosal barrier and function, and then damages the function and homeostasis of distant organs, leading to the occurrence of diseases [34]. At the phylum level, the abundance of species in the model group did not change significantly compared to the normal group, but at the genus level, there were significant changes. Therefore, it seems that the change in certain genera within these phyla has led to ecological imbalance. At the genus level, the relative abundance of *Faecalibaculum* in the model group was significantly lower than that in the normal group. In the Canadian Healthy Infant Longitudinal Development study, the authors compared the gut microbiota of 319 subjects. The results showed that infants at risk of asthma exhibited transient gut microbiota dysbiosis in the first 100 days of life. In these children at risk of asthma, the relative abundance of the bacterial genus *Faecalibacterium* was significantly reduced, and there was a decrease in acetate production and a disturbance in intestinal and liver metabolites [35]. In addition, Liu M et al. investigated the effects of Cangma Huadu granules on severe influenza pneumonia in a mouse model of H1N1-virus-induced acute lung injury. A higher abundance of *Faecalibaculum* was observed in the intestines of the Cangma Huadu treatment group [36]. In our study, the relative abundance of *Faecalitalea* in the IM group was significantly decreased compared to that in the IN group. Previous studies have reported that *Faecalitalea*, as a beneficial bacterium, can ferment D-glucose, sucrose, D-mannose, and raffinose, with butyrate being the main end product of metabolism [37]. Short-chain fatty acids play an important role in intestinal energy metabolism, anti-inflammation, and protection of the intestinal mucosal barrier [38]. Our findings were supported by the results of the LEfSe analysis, which showed that *Faecalibaculum* and *Faecalitalea* were significantly enriched in the normal group, while *Intestinimonas* was significantly enriched in the model group. Previous studies have found that tetrahydrocurcumin (THC) treatment reduced the relative abundance of the pro-inflammatory bacterium *Intestinimonas* in the gut of OVA-induced mice, thereby reducing Th2-mediated inflammation [39]. In previous studies on the expression profiles of intestinal mucosa in asthma, the authors found only overexpressions of innate immune genes in the ileum in the differential expression of ileum, transverse colon, and rectum biopsies [40]. The stability of the gut microbiota contributes to the intestinal mucosal barrier and nutrient uptake function, thereby maintaining immune balance [41]. Therefore, the overexpression of immune genes in the ileum may be related to changes in the intestinal microbiota, i.e., the ileal intestinal microbiota affects the mucosal immune system, thereby affecting the occurrence and development of CVA.

We continued our study of the composition of lung microbiota to further evaluate the microbiota changes in different ecological niches of the CVA model guinea pigs. Interestingly, there was no significant difference in the alpha diversity of lung microbiota between the normal and model groups, but beta diversity showed significant differences, indicating a significant change in the composition of lung microbiota. Avalos-Fernandez et al. systematically evaluated and meta-analyzed the alpha diversity of lung microbiota in asthma and chronic obstructive pulmonary disease patients, and half of the studies showed no significant difference in the alpha diversity index between the control and case groups [42]. When using the Shannon index, alpha diversity tended to be higher in healthy individuals than in severe asthmatic patients [43]. Further analysis of lung microbial composition showed a significant increase in *Tenericutes* in the model group, which is consistent with previous research. This study confirmed that *Tenericutes* is the top phylum of BALF microbiota in the *Mycoplasma* pneumoniae single infection group [44]. We also found that the ratio of *Proteobacteria* and *Cyanobacteria* in the lungs of the model group increased compared to the normal group. Previous research has shown that the overexpression of IL-13 in transgenic mice leads to enhanced eosinophilic inflammation and airway hyperresponsiveness, as well as increased proportions of *Proteobacteria* and *Cyanobacteria* in the lungs [45]. At the genus level, we observed a significant enrichment of *Mycoplasma* in the model group. *Mycoplasma* is a cell wall-free bacterium associated with various respiratory diseases and is increasingly recognized as an important pathogen in acute and chronic diseases. *Mycoplasma* may appear before asthma attacks, exacerbate asthma symptoms, or make asthma control more difficult [46]. In addition, *Mycoplasma* may trigger TH2 reactions and promote the development of atopic inflammatory reactions [47]. Therefore, our results support the hypothesis that *Mycoplasma* may play an important role in CVA disease. These results contribute to a deeper understanding of the relationship between microbiota and respiratory diseases and provide references for future clinical treatment and prevention.

Finally, we compared the microbiota of the CN and CM groups and performed alpha and beta diversity analyses. The results indicated that the Shannon index of the colon in the model group significantly increased, while the Chao1 index did not change significantly. This suggests that in the CVA model, the species richness of the colon microbiota did not significantly increase, but the complexity of the community did increase. Additionally, PCoA showed a partial overlap between the CN and CM groups, indicating fewer differences in community structure between them. Further analysis at the genus level revealed that compared to the CN group, the relative abundance of *Ruminococcus* and *Streptococcus* significantly increased in the CM group, while the relative abundance of *Faecalibaculum* and *Bacteroides* decreased. Previous studies have reported that Ephedra sinica polysaccharide (ESP) alleviates symptoms of mouse allergic asthma by regulating the intestinal microbiota and SCFA. ESP increased the relative proportion of *Bacteroides* and decreased that of *Ruminococcus* [48]. In patients with persistent atopic dermatitis, the level of *Streptococcus* is higher. The relative abundance of *Streptococcus* is positively correlated with the score of atopic dermatitis evaluation, and the persistent AD group shows a decrease in intestinal microbial functional genes associated with oxidative phosphorylation [49]. In addition, the decrease in *Faecalibaculum* is consistent with the results in the ileum, likely accompanied by a decrease in acetate production. LEfSe analysis revealed the enrichment of *Ruminococcaceae* and *Akkermansia* in the CM group. Previous studies have shown that the relative abundance of *Ruminococcaceae* and *Akkermansia* is positively correlated with Th2-related factors in asthma mice treated with THC. The relief of THC-mediated airway allergic inflammation depends on the regulation of the gut microbiota [39]. The gut microbiota can produce anti-inflammatory and immune-regulatory compounds, such as short-chain fatty acids and regulatory T cells, which can alleviate symptoms and inflammatory reactions in asthma patients [50]. In summary, changes in gut microbiota may be a key factor affecting the pathogenesis of CVA.

Interestingly, during our assessment of the microbiota of three ecological niches in CVA guinea pigs, we discovered that although the alpha diversity index of the ileum increased significantly, the number and diversity of species in the colon niche remained the highest. The PCoA analysis revealed a noteworthy observation that the community structure of the ileum and colon in the model group exhibited a similarity. As mentioned earlier, compared to the normal group, there was a significant change in the composition of the ileal microbiota in the CVA model group, while the difference in the colon was small. This suggests that the community structure of the ileum niche may be increasingly approaching that of the colon niche.

It is universally acknowledged that the deregulation of Th1/Th2 in CVA manifests predominantly as a Th2 immune response [51]. Cytokines such as IL-12 and IL-13 are deemed to play a pivotal role in the TH2-polarized immune response following inhalation of allergens. IL-12 has been demonstrated to trigger a TH2-polarized immune reaction in its absence [52]. The overproduction of IL-13 has been proven to induce many common features of allergic pathologies, such as airway hyperresponsiveness, eosinophilic inflammation, and excessive mucus secretion [53]. Previous research has shown that the imbalance in the proportion and function of Th1 and Th2 subgroups is the main pathogenic mechanism of asthma attacks, characterized by a decline in the function of Th1 cell subsets and a hyperfunction of Th2 cell subsets [54]. In alignment with these findings, our study further revealed a positive correlation between IL-13 and *Mycoplasma* in the lung, as well as *Ruminococcus* in the colon. Conversely, a negative correlation was observed between IL-13 and *Faecalibaculum* in the ileum. Moreover, IL-12 exhibited a negative correlation with pulmonary *Mycoplasma* and colonic *Ruminococcus*, while exhibiting a positive correlation with ileal *Faecalibaculum*. Nonetheless, further research is required to explore the potential relationship between Th1/Th2 cells and core microorganisms *Mycoplasma*, *Ruminococcus*, and *Faecalibaculum*.

## 4. Materials and Methods

### 4.1. Chemicals and Reagents

The Ovalbumin (OVA, ID: M0228A) and the aluminum hydroxide (Al(OH)_3_, ID: O1004A) compounds were purchased from Meilun Biotech Co., Ltd. (Dalian, China). The Guinea pig interleukin-12 (IL-12) kit (ID: MM-035902) and the guinea pig interleukin-13 (IL-13) kit (ID: MM-089802) were purchased from the Jiangsu Meimian Industrial Co., Ltd. (Yancheng, China). The Zymo Research BIOMICS DNA Microprep Kit (ID: D4301) and Zymoclean Gel Recovery Kit (ID: D4008) were obtained from Zymo Research Co., Ltd. (Irvine, CA, USA). The MegaFi Fidelity DNA Polymerase (ID: G896) was procured from Applied Biological Materials Inc. (Richmond, BC, Canada). The Nanopore 16S Barcoding Kit (ID: SQK-RAB204), Nanopore Ligation Sequencing Kit (ID: SQK-LSK109), and Nanopore Flow Cell Priming Kit (ID: EXP-FLP002) were acquired from Oxford Nanopore Technologies Co., Ltd. (Oxford, UK).

### 4.2. Animal

Twelve male guinea pigs, weighing between 220 and 250 g, were purchased from Yifengda Biotechnology Co., Ltd. (Xi’an, China). The experimental animal license number was SCXK (Shaanxi) 2020-003. These guinea pigs were being housed at the Institute of Laboratory Animals of Sichuan Academy of Medical Sciences & Sichuan Provincal People’s Hospital. The temperature was maintained between 20 and 26 °C, with a relative humidity of 40–70%, and alternating periods of 12 h of light and darkness. Three guinea pigs were randomly placed in each cage. They were provided with free access to food and water. 

### 4.3. Construction of CVA Guinea Pig Model

**Figure 12:**
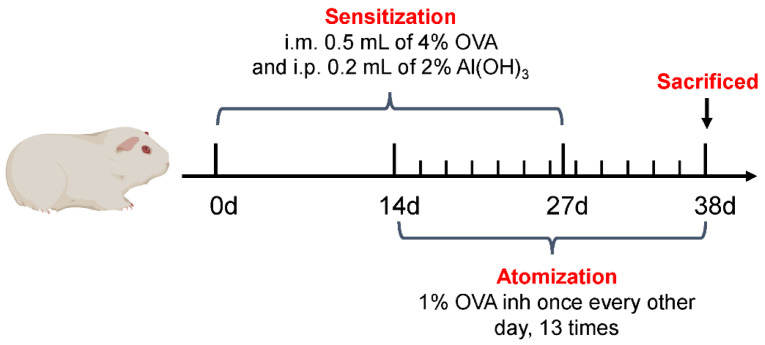
Establishment of a guinea pig model of cough variant asthma.

Guinea pigs were randomly divided into a normal group and a model group, with six guinea pigs in each group. After 10 days of adaptive feeding, the model group was sensitized with OVA and stimulated to prepare a CVA model (Figure 12), namely, intramuscular injection of 0.5 mL of 4% OVA solution on day 0 and day 27, and intraperitoneal injection of 0.2 mL of 2% Al(OH)_3_ suspension. The normal group was injected with the same amount of saline at the same time and by the same route. Starting from day 14, guinea pigs were placed in a 4 L sealed box and sprayed with 1% OVA solution at the maximum spray rate (3 mL/min) for 60 s, while the normal group was sprayed with physiological saline, inhaling once every other day, for a total of 13 times.

### 4.4. ELISA of Inflammatory Cytokines in BALF

Intraperitoneal injection of 2% sodium pentobarbital (50 mg/kg body weight) was used to anesthetize guinea pigs. The skin of the neck and chest was cut open along the midline of the anterior abdomen, and the trachea was dissected bluntly. Five milliliters of 4 °C saline was slowly injected twice into the tracheal intubation site to collect the bronchoalveolar lavage fluid (BALF). After centrifugation (3000 rpm/min, 10 min, 4 °C), the supernatant was collected and stored at −80 °C for further analysis. An enzyme-linked immunosorbent assay (ELISA) kit was used to measure IL-12 and IL-13 levels in BALF.

### 4.5. Analysis of 16S rDNA of Microbiota

The BALF, ileum, and colon contents were collected and preserved in sterile freezing tubes at −80 °C. Four samples were randomly chosen from each group and sent to Rhonin Biosciences Co., Ltd., Chengdu, China, for sequencing. Firstly, the DNA sample underwent purification. The gDNA was purified using the Zymo Research Biomics DNA Microprep Kit, and its integrity was verified through 0.8% agarose electrophoresis. The nucleic acid concentration was then measured using Tecan F200. PCR amplification was subsequently performed. Specific primers with index sequences were synthesized to amplify the full-length fragment of the 16S rDNA gene of the sample, in accordance with the sequencing region. The amplification primer sequences were as follows: Primer 5′-3′: 8F (5′-AGAGTTTGATCATGGCTCAG-3′) and 1492R (5′-CGGTTACCTTGTTACGACTT-3′). The PCR products were assessed by target fragment electrophoresis utilizing 1% agarose gel. The samples that passed the assay were retrieved by extracting the target bands using the Zymoclean Gel Recovery Kit and mixing them in equimolar amounts after quantification. Finally, the library was built using the Nanopore R9.4.1 library building kit, and the library was sequenced using the Nanopore GridION sequencer and subjected to real-time high-precision base calling.

Following the utilization of tools such as Qcat and NanoFILT to eliminate low-quality sequences and chimeras, we procured valuable data. We employed Kraken2 to annotate each sequence, and the number of species was tallied to create the operational taxonomic unit (OTU) table [55]. OTU is a clustering method that groups similar sequences into a smaller number of taxonomic units, with each OTU providing a representative sequence. This simplifies and clarifies the subsequent species annotation analysis. Through sequence comparison, the appropriate evolutionary model and reconstruction method are selected based on the difference between sequence and sequence characteristics, and an evolutionary tree is constructed [56]. The microbial community composition analysis, the alpha diversity, and beta diversity analysis were performed using R language (4.0.3). The differential species analysis was performed using Python (3.7.4).

### 4.6. Bioinformatics Analysis

The correlation between physicochemical factors and abundance species was calculated by pairwise Spearman rank correlation. Statistical analysis and plotting were performed using programming languages R (version 4.0.3), Python (version 3.7.4), and Java (version 8).

### 4.7. Statistical Analysis

The data generated in this study were statistically analyzed using IBM SPSS Statistics 27.0 software, and the results are presented as mean ± standard deviation ($\bar{x}$ ± SD). Graphs were generated using GraphPad Prism 8.0.1 software. Student’s *t*-test was used to compare the two groups. A *p*-value < 0.05 was considered statistically significant.

## 5. Conclusions

In this paper, we conducted a systematic analysis of the microbiota in the lung, ileum, and colon of normal and CVA guinea pigs and revealed the distinct differences among them. The ileal microbiota in the CVA model group was markedly dysbiotic, while the lung and colon microbiota also changed to some extent. Simultaneously, we observed that the ileal community structure in the model group tended to the colon. Moreover, we explored the potential association between Th1/Th2 cytokines and microbiota and identified some core microorganisms that may be implicated in CVA, such as *Mycoplasma*, *Ruminococcus*, and *Faecalibaculum*. The results of this study suggest that CVA development is closely related to the dysregulation of ileal, lung, and colon microbiota and the ensuing inflammatory changes in the lung. Currently, we have only detected the changes in the microbiota in CVA guinea pigs. In future research, we will investigate the function of core microorganisms to develop CVA disease modulation therapies based on microbiota.

## Data Availability

The microbiota sequencing data which support the findings in our study have been deposited into NCBI’s Sequence Read Archive under accession number PRJNA956819.
